# Imperfection Sensitivity of Nonlinear Vibration of Curved Single-Walled Carbon Nanotubes Based on Nonlocal Timoshenko Beam Theory

**DOI:** 10.3390/ma9090786

**Published:** 2016-09-21

**Authors:** Iman Eshraghi, Seyed K. Jalali, Nicola Maria Pugno

**Affiliations:** 1 School of Mechanical Engineering, University of Tehran, Tehran 1193653471, Iran; ieshraghi@ut.ac.ir; 2 Department of Mechanical Engineering, Kermanshah University of Technology, Kermanshah 6715685438, Iran; 3 Laboratory of Bio-Inspired & Graphene Nanomechanics, Department of Civil, Environmental and Mechanical Engineering, Università di Trento, via Mesiano, 77, Trento 38123, Italy; nicola.pugno@unitn.it; 4 Center for Materials and Microsystems, Fondazione Bruno Kessler—via Sommarive 18, Povo, Trento 38123, Italy; 5 School of Engineering & Materials Science, Queen Mary University of London—Mile End Road, London E1 4NS, UK

**Keywords:** nonlinear vibration, imperfection, curved SWCNT, nonlocal theory, differential quadrature method (DQ)

## Abstract

Imperfection sensitivity of large amplitude vibration of curved single-walled carbon nanotubes (SWCNTs) is considered in this study. The SWCNT is modeled as a Timoshenko nano-beam and its curved shape is included as an initial geometric imperfection term in the displacement field. Geometric nonlinearities of von Kármán type and nonlocal elasticity theory of Eringen are employed to derive governing equations of motion. Spatial discretization of governing equations and associated boundary conditions is performed using differential quadrature (DQ) method and the corresponding nonlinear eigenvalue problem is iteratively solved. Effects of amplitude and location of the geometric imperfection, and the nonlocal small-scale parameter on the nonlinear frequency for various boundary conditions are investigated. The results show that the geometric imperfection and non-locality play a significant role in the nonlinear vibration characteristics of curved SWCNTs.

## 1. Introduction

Nanostructures have attracted more attention in recent years due to their superior mechanical, electrical, and chemical characteristics [1]. These properties have led to widespread applications of these structures in nano-electro-mechanical-system (NEMS) devices. This growth in development and use of nanostructures necessitates designing, fabricating and characterizing their mechanical behavior. The literature reveals that carbon nanotubes (CNTs) have the potential to be used in these devices. Many of the NEMS devices like oscillators and mass measuring sensors use the vibration characteristics of the CNTs [2,3,4]. This has led to a remarkable number of studies conducted on the characterization of vibration behavior of CNTs [5,6,7,8,9,10]. A review including modeling, simulation and application of vibration behavior of CNTs can be found in [11].

Various methods are utilized to model and analyze the behavior of nano-beams. Among them, nonlocal elasticity of Eringen [12], as a non-classical theory of elasticity which considers the scale effect, has been widely used for mathematical continuum modeling and analysis of bending, buckling and vibration of nanostructures. In this way, known classical theories of beams like Euler-Bernoulli or Timoshenko beam theory can be adopted to model the displacement field of a CNT and nonlocal theory may be employed to modify constitutive stress-strain relationships to take small-scale effect into account. There has been extensive study on modeling and analysis of CNTs using this theory [13,14,15]. Wang and Varadan [16] investigated the free vibration of both single-walled and double-walled nanotubes via nonlocal elasticity. The small-scale effects on the vibration characteristics of carbon nanotubes were explicitly derived in their work, and effects of the length and diameter scale on the vibrational frequency were revealed. Another study by Lu et al. [17] investigated application of nonlocal Euler and Timoshenko beam models for vibration and wave propagation of CNTs and provided the effects of small-scale parameter on the free vibration frequency. Murmu and Pradhan [18] studied thermo-mechanical vibration of a SWCNT embedded in an elastic medium based on nonlocal elasticity theory. They used the DQ method to numerically find thermal vibration response of CNTs. Furthermore, influences of nonlocal small-scale effects, temperature change and elastic medium constant on the frequency values of various modes were investigated. The free vibration response of SWCNTs using the nonlocal elasticity based on Euler–Bernoulli, Timoshenko and Reddy beam theory for various boundary conditions was investigated by Ansari and Sahmani [19]. They obtained fundamental frequencies of nanotubes with different chiralities by implementing molecular dynamics (MD) simulation and comparing the output of their simulations with the results obtained by the nonlocal beam models to identify appropriate values of nonlocal parameter for various types of chirality and boundary conditions.

In spite of significance of nonlinear vibrational response of CNTs, the number of studies which consider large amplitude vibrations are limited. Reddy [20,21] used nonlocal elasticity theory and von Kármán nonlinear strain-displacement relations to reformulate beam and plate theories for analysis of bending, buckling and vibration behavior of these nanostructures. Yang et al. [22] developed nonlinear free vibration equations of SWCNTs using nonlocal Timoshenko beam theory and used DQ procedure to discretize the governing equations. They obtained elastic modulus through molecular mechanics simulation. The influences of nonlocal parameter, length and radius of the nano-beam and type of supports on the nonlinear free vibration characteristics were investigated by using an iterative procedure. Nonlinear vibration analysis of double-walled carbon nanotubes based on nonlocal elasticity theory considering the von Kármán type geometric nonlinearity was studied by Fang et al. [23]. It was shown that nonlocal parameter, aspect ratio and surrounding elastic medium have more effect on the nonlinear non-coaxial vibration than the coaxial vibration of the nanotubes.

All the aforesaid works modeled CNTs as straight beams. However, an examination of the studies concerned with fabrication procedures of CNTs reveals that perfectly straight CNTs without any deviation in geometry and orientation are very difficult to achieve [5]. In a historical view of classical beam theories, these deviations could be modeled as an initial geometric imperfection. Initial geometric imperfection refers to globally or locally unavoidable deviations between the actual shape and desired shape of the structure [24]. It is reported that such imperfections have remarkable effects on nonlinear vibration frequencies of structures [25,26]. There has been quite a few works considering the effects of geometrical imperfection in continuum modeling of CNTs. Farshidianfar and Soltani [27] based on the nonlocal continuum theory analyzed transverse vibration of a curved single-walled carbon nanotube conveying a fluid. They discussed the effects of the flow velocity, nonlocal parameter, the stiffness of the elastic foundation, and the boundary conditions in detail. Ouakad and Younis [28] reported the natural frequencies and mode shapes of initially curved carbon nanotube resonators under electric excitation. The variation of natural frequencies and mode shapes with amplitude of imperfection and the DC electrostatic load using a multimode Galerkin procedure were investigated.

The aim of this paper is to compute the imperfection sensitivity of large amplitude vibration of curved SWCNTs based on a nonlocal Timoshenko beam model. To include geometric nonlinearities, von Kármán type of strain-displacement relation is adopted for small strain and moderate rotations of the beam. An iterative method [22] is employed to solve the discretized eigenvalue equation of the free vibration of the beam. Extensive numerical results are presented to investigate the influence of amplitude and configuration of the geometric imperfection, boundary conditions and the nonlocal small-scale parameter on vibration behavior of a curved SWCNT.

## 2. Problem Formulation

### 2.1. Timoshenko Beam Model for Vibration of Curved Single-Walled Carbon Nanotubes (SWCNTs)

**Figure 1:**
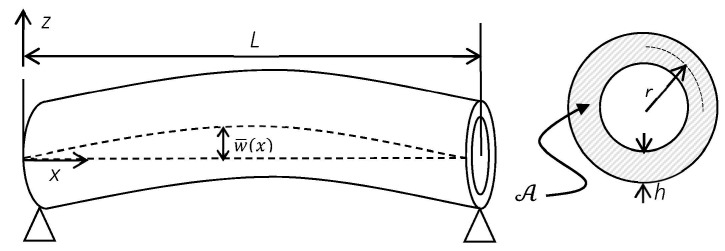
An initially curved beam with annular cross-section representing a SWCNT.

The curved SWCNT as a Timoshenko beam model with length *L*, constant cross section, A, mean wall radius *r* and thickness *h*, is illustrated in Figure 1. $\bar{w}\left(x\right)$ denotes the typical initial geometric imperfection of the curved SWCNT.

By using Timoshenko beam theory, displacement components along *x*- and *z*- axes are given by

(1) $$\begin{array}{c} u\left(x,z,t\right)=u^{0}\left(x,t\right)+z\phi \left(x,t\right)\\ w\left(x,z,t\right)=w^{0}\left(x,t\right)+\bar{w}\left(x\right) \end{array}$$

where $u^{0}\left(x,t\right)$ and $w^{0}\left(x,t\right)$ are longitudinal and transversal displacements of the straight beam axis, ϕ(x,t), denotes rotation of the cross-section with respect to the straight beam axis and t represents time. For the case of large amplitude vibration, based on nonlinear strain-displacement relations of von Kármán type, the strain components at any point are expressed as [29]:

(2) $$\begin{array}{c} \varepsilon^{xx}=u_{,x}^{0}+\frac{1}{2}{\left(w_{,x}^{0}\right)}^{2}+w_{,x}^{0}{\bar{w}}_{,x}+z\phi_{,x}\\ \gamma^{xz}=w_{,x}^{0}+\phi \end{array}$$

where $\varepsilon^{xx}$ is the normal strain, and $\gamma^{xz}$ is the shear strain. Note that ${}_{,x}$ represents differentiation with respect to *x*. Using the principle of virtual displacements [20] and taking into account geometric initial imperfection, the Euler-Lagrange equations for the beam are given by

(3) $$\begin{array}{c} N_{,x}+f=I_{0}{\ddot{u}}^{0}\\ Q_{,x}+{\left[N\left(w_{,x}^{0}+{\bar{w}}_{,x}\right)\right]}_{,x}+q=I_{0}{\ddot{w}}^{0}\\ M_{,x}-Q=I_{2}\ddot{\phi } \end{array}$$

And the corresponding immovable boundary conditions at the ends of the beam (*x = 0*, *L*) are

(4) $$\begin{array}{ccc} u^{0}=0,\;w^{0}=0,\;\phi =0 & & \text{Clamped End}\\ u^{0}=0,\;w^{0}=0,\;M=0 & & \text{Hinged End} \end{array}$$

In Equation (3) a dot above each quantity represents temporal differentiation. f and q are externally applied distributed forces (per length unit) on the beam in the *x*- and *z*- directions, respectively. $I_{0}$ and $I_{2}$ are mass moments of inertia of the beam section given by

(5) $$\begin{array}{c} I_{0}=\overset{}{\underset{A}{\int }}\rho \left(x,z\right)dA\\ I_{2}=\overset{}{\underset{A}{\int }}z^{2}\rho \left(x,z\right)\;dA \end{array}$$

where ρ(x,z) is the beam material density. The resultant moment *M*, normal force *N*, and shear force *Q* can be calculated by integrating the corresponding axial (σ) and in-plane shear stress (τ) components on the cross-section area as

(6) $$\begin{array}{c} N=\overset{}{\underset{A}{\int }}\sigma^{xx}\;dA\\ M=\overset{}{\underset{A}{\int }}z\;\sigma^{xx}\;dA\\ Q=\overset{}{\underset{A}{\int }}\tau^{xz}\;dA \end{array}$$

### 2.2. Nonlocal Theory of Nano-Beams

Based on Eringen’s nonlocal elasticity theory, the state of stress at a point in a material is a function of strain field at every point in the continuum [12]. The integral form of the nonlocal theory that defines stress in each point in the material is stated as

(7) $$\sigma =\overset{}{\underset{V}{\int }}\chi \left(\left|Y-X\right|,\lambda \right)S\left(Y\right)dY$$

where X is the location of the reference point and χ(|Y−X|,τ) is a function that imposes the effect of strain at point Y into the nonlocal constitutive equation. |Y−X| denotes the distance between two points and λ is the parameter for small-scale factor. S(Y) is the classical stress tensor that is related to local strain field for a Hookean solid using fourth-order elastic moduli tensor C(X) according to the following inner product operation of tensors

(8) S(X)=C(X):ε(X)

Equivalent differential form of Equation (7) can be represented by [20]:

(9) $$Ɫ\left(\sigma \right)=S\;,\;Ɫ=1-{\tilde{\mu }}^{2}\nabla^{2}\;\text{and}\;\tilde{\mu }=e_{0}a_{0}$$

where $e_{0}$ is a material constant and $a_{0}$ is an internal characteristic length [30] and $\nabla^{2}$ is the Laplacian operator which is equal to $d^{2}/dx^{2}$ for a one-dimensional case. Thus the nonlocal constitutive relations of a nano-beam can be written as

(10) $$\begin{array}{c} \sigma^{\text{xx}}-{\tilde{\mu }}^{2}\sigma_{,\text{xx}}^{\text{xx}}=E\varepsilon^{\text{xx}}\\ \tau^{\text{xz}}-{\tilde{\mu }}^{2}\tau_{,\text{xx}}^{\text{xz}}=G\gamma^{\text{xz}} \end{array}$$

where E and G are the Young’s modulus and shear modulus of the nano-beam, respectively. 

### 2.3. Derivation of Nonlocal Governing Equations of Motion

Now, integrating Equation (10) on the cross-section area with the assumption of constant material properties and using Equations (2) and (6) for the definition of strain components and resultant forces, yields

(11) $$\begin{array}{c} N-{\tilde{\mu }}^{2}N_{,xx}=EA\left(u_{,x}^{0}+\frac{1}{2}{\left(w_{,x}^{0}\right)}^{2}+w_{,x}^{0}{\bar{w}}_{,x}\right)\\ M-{\tilde{\mu }}^{2}M_{,xx}=Eℐ\phi_{,x}\\ Q-{\tilde{\mu }}^{2}Q_{,xx}=K_{s}GA\left(w_{,x}^{0}+\phi \right) \end{array}$$

where ℐ is the area moment of inertia of the beam cross-section $\left(ℐ=\int_{A}^{}z^{2}dA\right)$ and $K_{s}$ is the shear correction factor. Substituting Equation (3) into Equation (11) and neglecting distributed external forces for the problem of free vibration, stress resultants can be obtained in terms of displacement components as:

(12) $$\begin{array}{c} N=EA\left(u_{,x}^{0}+\frac{1}{2}{\left(w_{,x}^{0}\right)}^{2}+w_{,x}^{0}{\bar{w}}_{,x}\right)+{\tilde{\mu }}^{2}I_{0}{\ddot{u}}_{,x}^{0}\\ M=Eℐ\phi_{,x}+{\tilde{\mu }}^{2}\left\{I_{0}{\ddot{w}}^{0}-{\left[N\left(w_{,x}^{0}+{\bar{w}}_{,x}\right)\right]}_{,x}+I_{2}{\ddot{\phi }}_{,x}\right\}\\ Q=K_{s}GA\left(w_{,x}^{0}+\phi \right)+{\tilde{\mu }}^{2}\left\{I_{0}{\ddot{w}}_{,x}^{0}-{\left[N\left(w_{,x}^{0}+{\bar{w}}_{,x}\right)\right]}_{,xx}\right\} \end{array}$$

Thus, the equations of motion for the nonlocal Timoshenko beam considering nonlinear strain-displacement relations and initial geometric imperfection can be obtained by back substitution of Equation (12) into Equation (3).

(13) $$\begin{array}{cc} EA & \left(u_{,xx}^{0}+w_{,x}^{0}w_{,xx}^{0}+w_{,x}^{0}{\bar{w}}_{,xx}^{0}+w_{,xx}^{0}{\bar{w}}_{,x}^{0}\right)=I_{0}\left({\ddot{u}}^{0}-\mu {\ddot{u}}_{,xx}^{0}\right)\\ K_{s}GA\left(w_{,xx}^{0}+\phi_{,x}\right) & +\Lambda_{1}-{\tilde{\mu }}^{2}\Lambda_{2}\\ & =I_{0}\left({\ddot{w}}^{0}-{\tilde{\mu }}^{2}{\ddot{w}}_{,xx}^{0}\right)\\ & -I_{0}{\tilde{\mu }}^{2}\{{\ddot{u}}_{,xx}^{0}\left(w_{,x}^{0}+{\bar{w}}_{,x}\right)+{\ddot{u}}_{,x}^{0}\left(w_{,xx}^{0}+{\bar{w}}_{,xx}\right)\\ & -{\tilde{\mu }}^{2}[{\ddot{u}}_{,xxxx}^{0}\left(w_{,x}^{0}+{\bar{w}}_{,x}\right)+{\ddot{u}}_{,xxx}^{0}\left(w_{,xx}^{0}+{\bar{w}}_{,xx}\right)+{\ddot{u}}_{,xx}^{0}\left(w_{,xxx}^{0}+{\bar{w}}_{,xxx}\right)\\ & +{\ddot{u}}_{,x}^{0}\left(w_{,xxxx}^{0}+{\bar{w}}_{,xxxx}\right)]\}\\ & Eℐ\phi_{,xx}-K_{s}GA\left(w_{,x}^{0}+\phi \right)=I_{2}\left(\ddot{\phi }-{\tilde{\mu }}^{2}{\ddot{\phi }}_{,xx}\right) \end{array}$$

where

(14) $$\begin{array}{cc} \Lambda_{1}=EA[u_{,\text{xx}}^{0}{\bar{w}}_{,x} & +u_{,x}^{0}{\bar{w}}_{,\text{xx}}+w_{,\text{xx}}^{0}\left(u_{,x}^{0}+\frac{3}{2}w_{,x}^{0}w_{,x}^{0}+3w_{,x}^{0}{\bar{w}}_{,x}+{\bar{w}}_{,x}{\bar{w}}_{,x}\right)\\ & +w_{,x}^{0}\left(u_{,\text{xx}}^{0}+\frac{3}{2}w_{,x}^{0}{\bar{w}}_{,\text{xx}}+2{\bar{w}}_{,\text{xx}}{\bar{w}}_{,x}\right)]\\ \Lambda_{2}=EA[u_{,xxxx}^{0}{\bar{w}}_{,x} & +3u_{,xxx}^{0}{\bar{w}}_{,xx}+3u_{,xx}^{0}{\bar{w}}_{,xxx}+u_{,x}^{0}{\bar{w}}_{,xxxx}\\ & +w_{,xxxx}^{0}\left(u_{,x}^{0}+\frac{3}{2}w_{,x}^{0}w_{,x}^{0}+3w_{,x}^{0}{\bar{w}}_{,x}+{\bar{w}}_{,x}{\bar{w}}_{,x}\right)\\ & +w_{,xxx}^{0}\left(3u_{,xx}^{0}+9w_{,xx}^{0}w_{,x}^{0}+9w_{,xx}^{0}{\bar{w}}_{,x}+9w_{,x}^{0}{\bar{w}}_{,xx}+6{\bar{w}}_{,xx}{\bar{w}}_{,x}\right)\\ & +w_{,xx}^{0}(3u_{,xxx}^{0}+3w_{,xx}^{0}w_{,xx}^{0}+9w_{,xx}^{0}{\bar{w}}_{,xx}+9w_{,x}^{0}{\bar{w}}_{,xxx}+6{\bar{w}}_{,xx}{\bar{w}}_{,xx}\\ & +6{\bar{w}}_{,xxx}{\bar{w}}_{,x})+w_{,x}^{0}\left(u_{,xxxx}^{0}+\frac{3}{2}w_{,x}^{0}{\bar{w}}_{,xxxx}+2{\bar{w}}_{,xxxx}{\bar{w}}_{,x}+6{\bar{w}}_{,xxx}{\bar{w}}_{,xx}\right)] \end{array}$$

The following non-dimensional parameters are introduced to make equations of motion dimensionless:

(15) $$\begin{array}{c} \xi =\frac{x}{L},\;\alpha =\frac{h}{L},\;\left(u^{*},w^{*},{\bar{w}}^{*}\right)=\frac{\left(u^{0},w^{0},\bar{w}\right)}{h},\;\phi =\phi ,\;\left(I_{0}^{*},I_{2}^{*}\right)=\left(\frac{I_{0}}{I_{0}},\frac{I_{2}}{I_{0}h^{2}}\right),\;\left(D_{0}^{*},D_{2}^{*},G^{*}\right)=\left(\frac{EA}{EA},\frac{Eℐ}{EAh^{2}},\frac{K_{s}GA}{EA}\right),\;\mu =\frac{\tilde{\mu }}{L},\\ t^{*}=\frac{t}{L}\sqrt{\frac{EA}{I_{0}}} \end{array}$$

Noting that now a dot superscript denotes differentiation with respect to non-dimensional time ($t^{*}$) and ${}_{,\xi }$ represents differentiation with respect to non-dimensional parameter, ξ. μ is called the nonlocal parameter. Thus, Equation (13) and the boundary conditions, Equation (4), can be rewritten in the following form.

(16) $$\begin{array}{cc} D_{0}^{*}[u_{,\xi \xi }^{*} & +\alpha \left(w_{,\xi }^{*}w_{,\xi \xi }^{*}+w_{,\xi }^{*}{\bar{w}}_{,\xi \xi }^{*}+w_{,\xi \xi }^{*}{\bar{w}}_{,\xi }^{*}\right)]=I_{0}^{*}\left({\ddot{u}}^{*}-\mu^{*}{\ddot{u}}_{,\xi \xi }^{*}\right)\\ G^{*}\left(w_{,\xi \xi }^{*}+\frac{1}{\alpha }\phi_{,\xi }\right) & +\Lambda_{1}^{*}-\mu^{2}\Lambda_{2}^{*}\\ & =I_{0}^{*}\left({\ddot{w}}^{*}-\mu^{2}{\ddot{w}}_{,\xi \xi }^{*}\right)\\ & -I_{0}^{*}\mu^{2}\alpha \{{\ddot{u}}_{,\xi \xi }^{*}\left(w_{,\xi }^{*}+{\bar{w}}_{,\xi }^{*}\right)+{\ddot{u}}_{,\xi }^{*}\left(w_{,\xi \xi }^{*}+{\bar{w}}_{,\xi \xi }^{*}\right)\\ & -\mu^{2}[{\ddot{u}}_{,\xi \xi \xi \xi }^{*}\left(w_{,\xi }^{*}+{\bar{w}}_{,\xi }^{*}\right)+{\ddot{u}}_{,\xi \xi \xi }^{*}\left(w_{,\xi \xi }^{*}+{\bar{w}}_{,\xi \xi }^{*}\right)+{\ddot{u}}_{,\xi \xi }^{*}\left(w_{,\xi \xi \xi }^{*}+{\bar{w}}_{,\xi \xi \xi }^{*}\right)\\ & +{\ddot{u}}_{,\xi }^{*}\left(w_{,\xi \xi \xi \xi }^{*}+{\bar{w}}_{,\xi \xi \xi \xi }^{*}\right)]\}\\ & D_{2}^{*}\phi_{,\xi \xi }-\frac{1}{\alpha }G^{*}\left(w_{,\xi }^{*}+\frac{1}{\alpha }\phi \right)=I_{2}^{*}\left(\ddot{\Phi }-\mu^{2}{\ddot{\phi }}_{,\xi \xi }\right) \end{array}$$

(17) $$\begin{array}{cc} \Lambda_{1}^{*}=D_{0}^{*}\alpha \;\{u_{,\xi \xi }^{*}{\bar{w}}_{,\xi }^{*} & +u_{,\xi }^{*}{\bar{w}}_{,\xi \xi }^{*}+w_{,\xi \xi }^{*}\left[u_{,\xi }^{*}+\alpha \left(\frac{3}{2}w_{,\xi }^{*}w_{,\xi }^{*}+3w_{,\xi }^{*}{\bar{w}}_{,\xi \xi }^{*}+{\bar{w}}_{,\xi }^{*}{\bar{w}}_{,\xi }^{*}\right)\right]\\ & +w_{,\xi }^{*}\left[u_{,\xi \xi }^{*}+\alpha \left(\frac{3}{2}w_{,\xi }^{*}{\bar{w}}_{,\xi \xi }^{*}+2{\bar{w}}_{,\xi \xi }^{*}{\bar{w}}_{,\xi }^{*}\right)\right]\}\\ \Lambda_{2}^{*}=D_{0}^{*}\alpha \;\{u_{,\xi \xi \xi \xi }^{*}{\bar{w}}_{,\xi }^{*} & +3u_{,\xi \xi \xi }^{*}{\bar{w}}_{,\xi \xi }^{*}+3u_{,\xi \xi }^{*}{\bar{w}}_{,\xi \xi \xi }^{*}+u_{,\xi }^{*}{\bar{w}}_{,\xi \xi \xi \xi }^{*}\\ & +w_{,\xi \xi \xi \xi }^{*}\left[u_{,\xi }^{*}+\alpha \left(\frac{3}{2}w_{,\xi }^{*}w_{,\xi }^{*}+3w_{,\xi }^{*}{\bar{w}}_{,\xi }^{*}+{\bar{w}}_{,\xi }^{*}{\bar{w}}_{,\xi }^{*}\right)\right]\\ & +w_{,\xi \xi \xi }^{*}\left[3u_{,\xi \xi }^{*}+\alpha \left(9w_{,\xi \xi }^{*}w_{,\xi }^{*}+9w_{,\xi \xi }^{*}{\bar{w}}_{,\xi }^{*}+9w_{,\xi }^{*}{\bar{w}}_{,\xi \xi }^{*}+6{\bar{w}}_{,\xi \xi }^{*}{\bar{w}}_{,\xi }^{*}\right)\right]\\ & +w_{,\xi \xi }^{*}[3u_{,\xi \xi \xi }^{*}\\ & +\alpha \left(3w_{,\xi \xi }^{*}w_{,\xi \xi }^{*}+9w_{,\xi \xi }^{*}{\bar{w}}_{,\xi \xi }^{*}+9w_{,\xi }^{*}{\bar{w}}_{,\xi \xi \xi }^{*}+6{\bar{w}}_{,\xi \xi }^{*}{\bar{w}}_{,\xi \xi }^{*}+6{\bar{w}}_{,\xi \xi \xi }^{*}{\bar{w}}_{,\xi }^{*}\right)]\\ & +w_{,\xi }^{*}\left[u_{,\xi \xi \xi \xi }^{*}+\alpha \left(\frac{3}{2}w_{,\xi }^{*}{\bar{w}}_{,\xi \xi \xi \xi }^{*}+2{\bar{w}}_{,\xi \xi \xi \xi }^{*}{\bar{w}}_{,\xi }^{*}+6{\bar{w}}_{,\xi \xi \xi }^{*}{\bar{w}}_{,\xi \xi }^{*}\right)\right]\} \end{array}$$

Clamped end:

(18) $$u^{*}=w^{*}=\phi =0$$

Hinged end:

(19) $$\begin{array}{c} u^{*}=w^{*}=0\\ \begin{array}{cc} M=D_{2}^{*}\Phi_{,\xi }-D_{0}^{*}\mu^{2} & [{\bar{w}}_{,\xi }^{*}u_{,\xi \xi }^{*}+{\bar{w}}_{,\xi \xi }^{*}u_{,\xi }^{*}+w_{,\xi }^{*}\left(u_{,\xi \xi }^{*}+\frac{3}{2}\alpha \;w_{,\xi }^{*}{\bar{w}}_{,\xi \xi }^{*}+2\alpha {\bar{w}}_{,\xi \xi }^{*}{\bar{w}}_{,\xi }^{*}\right)\\ & +w_{,\xi \xi }^{*}\left(u_{,\xi }^{*}+\frac{3}{2}\alpha \;w_{,\xi }^{*}w_{,\xi }^{*}+3\alpha \;w_{,\xi }^{*}{\bar{w}}_{,\xi }^{*}+\alpha {\bar{w}}_{,\xi }^{*}{\bar{w}}_{,\xi }^{*}\right)]+I_{2}^{*}\mu^{2}{\ddot{\phi }}_{,\xi }\\ & -I_{0}^{*}\mu^{4}{\ddot{u}}_{,\xi \xi }^{*}\left(w_{,\xi }^{*}+{\bar{w}}_{,\xi }^{*}\right)-I_{0}^{*}\mu^{4}{\ddot{u}}_{,\xi }^{*}\left(w_{,\xi \xi }^{*}+{\bar{w}}_{,\xi \xi }^{*}\right)+\frac{1}{\alpha }\mu^{2}I_{0}^{*}{\ddot{w}}^{*}=0 \end{array} \end{array}$$

### 2.4. Differential Quadrature Solution Procedure

Application of the DQ method is suggested by many researchers for solving eigenvalue problems [31,32]. Indeed, this approach is based on polynomial approximation of a function at any location in the domain. Then partial derivatives of that function with respect to a domain coordinate are approximated by weighted summation of its values at all selected discrete points in the domain. Thus for the one-dimensional nano-beam considered here, displacement and rotation components and their *n*th derivatives may be approximated by [33]

(20) $$\left\{u^{*},\;w^{*},\;\phi \right\}=\overset{R}{\underset{q=1}{\sum }}L_{q}\left(\xi \right)\left\{u_{q}^{*},\;w_{q}^{*},\;\phi_{q}\right\}$$

(21) $${\left\{u_{,\xi^{n}}^{*},\;w_{,\xi^{n}}^{*}\;,\;\phi_{,\xi^{n}}\right\}|}_{\xi =\xi_{p}}=\overset{R}{\underset{q=1}{\sum }}C_{pq}^{n}\left\{u_{q}^{*},\;w_{q}^{*},\;\phi_{q}\right\},\;n=1,\;2,\;\ldots ,\;R-1,\;p=1,\;2,\;\ldots ,\;R$$

where R is the number of sampling grid points in the domain 0≤ξ≤1, $L_{q}\left(\xi \right)$ is the Lagrange interpolation polynomials and ${}_{,\xi^{n}}$ represents *n*th derivative with respect to ξ. The Chebyshev-Gauss-Lobatto grid points are used in this study and are given by [33]:

(22) $$\xi_{i}=\frac{1}{2}\left(1-cos\frac{\pi i}{R-1}\right),\;i=0,\;1,\ldots ,R-1$$

Recursive formula for weighting coefficients, $C_{pq}^{n}$, can be found in [33]. Introduction of DQ approximation of spatial derivatives of displacement and rotation components, Equations (20) and (21), into Equation (16) yields

(23) $$\begin{array}{cc} D_{0}^{*}[\overset{R}{\underset{q=1}{\sum }}C_{pq}^{\left(2\right)}u_{q}^{*} & +\alpha (\overset{R}{\underset{q=1}{\sum }}C_{pq}^{\left(1\right)}w_{q}^{*}\overset{R}{\underset{q=1}{\sum }}C_{pq}^{\left(2\right)}w_{q}^{*}+{{\bar{w}}_{,\xi \xi }^{*}|}_{\xi =\xi_{p}}\overset{R}{\underset{q=1}{\sum }}C_{pq}^{\left(1\right)}w_{q}^{*}\\ & +{{\bar{w}}_{,\xi }^{*}|}_{\xi =\xi_{p}}\overset{R}{\underset{q=1}{\sum }}C_{pq}^{\left(2\right)}w_{q}^{*})]=I_{0}^{*}\left({{\ddot{u}}^{*}|}_{\xi =\xi_{p}}-\mu^{2}\overset{R}{\underset{q=1}{\sum }}C_{pq}^{\left(2\right)}{\ddot{u}}_{q}^{*}\right)\\ G^{*}(\overset{R}{\underset{q=1}{\sum }}C_{pq}^{\left(2\right)}w_{q}^{*} & +\frac{1}{\alpha }\overset{R}{\underset{q=1}{\sum }}C_{pq}^{\left(1\right)}\Phi_{q}^{*})+\Lambda_{1}^{*}-\mu^{2}\Lambda_{2}^{*}\\ & =I_{0}^{*}\left({{\ddot{w}}^{*}|}_{\xi =\xi_{p}}-\mu^{2}\overset{R}{\underset{q=1}{\sum }}C_{pq}^{\left(2\right)}{\ddot{w}}_{q}^{*}\right)\\ & -I_{0}^{*}\mu^{2}\alpha \{\overset{R}{\underset{q=1}{\sum }}C_{pq}^{\left(2\right)}{\ddot{u}}_{q}^{*}\left({{\bar{w}}_{,\xi }^{*}|}_{\xi =\xi_{p}}+\overset{R}{\underset{q=1}{\sum }}C_{pq}^{\left(1\right)}w_{q}^{*}\right)\\ & +\overset{R}{\underset{q=1}{\sum }}C_{pq}^{\left(1\right)}{\ddot{u}}_{q}^{*}\left({{\bar{w}}_{,\xi \xi }^{*}|}_{\xi =\xi_{p}}+\overset{R}{\underset{q=1}{\sum }}C_{pq}^{\left(2\right)}w_{q}^{*}\right)\\ & -\mu^{2}[\overset{R}{\underset{q=1}{\sum }}C_{pq}^{\left(4\right)}{\ddot{u}}_{q}^{*}\left({{\bar{w}}_{,\xi }^{*}|}_{\xi =\xi_{p}}+\overset{R}{\underset{q=1}{\sum }}C_{pq}^{\left(1\right)}w_{q}^{*}\right)\\ & +\overset{R}{\underset{q=1}{\sum }}C_{pq}^{\left(3\right)}{\ddot{u}}_{q}^{*}\left({{\bar{w}}_{,\xi \xi }^{*}|}_{\xi =\xi_{p}}+\overset{R}{\underset{q=1}{\sum }}C_{pq}^{\left(2\right)}w_{q}^{*}\right)\\ & +\overset{R}{\underset{q=1}{\sum }}C_{pq}^{\left(2\right)}{\ddot{u}}_{q}^{*}\left({{\bar{w}}_{,\xi \xi \xi }^{*}|}_{\xi =\xi_{p}}+\overset{R}{\underset{q=1}{\sum }}C_{pq}^{\left(3\right)}w_{q}^{*}\right)\\ & +\overset{R}{\underset{q=1}{\sum }}C_{pq}^{\left(1\right)}{\ddot{u}}_{q}^{*}\left({{\bar{w}}_{,\xi \xi \xi \xi }^{*}|}_{\xi =\xi_{p}}+\overset{R}{\underset{q=1}{\sum }}C_{pq}^{\left(4\right)}w_{q}^{*}\right)]\}\\ D_{2}^{*}\overset{R}{\underset{q=1}{\sum }}C_{pq}^{\left(2\right)}\phi_{q}^{*}-\frac{1}{\alpha }G^{*} & \left(\overset{R}{\underset{q=1}{\sum }}C_{pq}^{\left(1\right)}w_{q}^{*}+\frac{1}{\alpha }{\phi |}_{\xi =\xi_{p}}\right)=I_{2}^{*}\left({\ddot{\Phi }|}_{\xi =\xi_{p}}-\mu^{2}\overset{R}{\underset{q=1}{\sum }}C_{pq}^{\left(2\right)}{\ddot{\phi }}_{q}^{*}\right) \end{array}$$

where

(24) $$\begin{array}{cc} \Lambda_{1}^{*}=D_{0}^{*}\alpha \;\{{{\bar{w}}_{,\xi }^{*}|}_{\xi =\xi_{p}} & \overset{R}{\underset{q=1}{\sum }}C_{pq}^{\left(2\right)}u_{q}^{*}+{{\bar{w}}_{,\xi \xi }^{*}|}_{\xi =\xi_{p}}\overset{R}{\underset{q=1}{\sum }}C_{pq}^{\left(1\right)}u_{q}^{*}\\ & +\overset{R}{\underset{q=1}{\sum }}C_{pq}^{\left(2\right)}w_{q}^{*}[\overset{R}{\underset{q=1}{\sum }}C_{pq}^{\left(1\right)}u_{q}^{*}+\frac{3}{2}\alpha {\left(\overset{R}{\underset{q=1}{\sum }}C_{pq}^{\left(1\right)}w_{q}^{*}\right)}^{2}\\ & +3\alpha {{\bar{w}}_{,\xi \xi }^{*}|}_{\xi =\xi_{p}}\overset{R}{\underset{q=1}{\sum }}C_{pq}^{\left(1\right)}w_{q}^{*}+\alpha {\left({{\bar{w}}_{,\xi }^{*}|}_{\xi =\xi_{p}}\right)}^{2}]\\ & +{{\bar{w}}_{,\xi }^{*}|}_{\xi =\xi_{p}}[\overset{R}{\underset{q=1}{\sum }}C_{pq}^{\left(2\right)}u_{q}^{*}\\ & +\alpha \left(\frac{3}{2}{{\bar{w}}_{,\xi \xi }^{*}|}_{\xi =\xi_{p}}\overset{R}{\underset{q=1}{\sum }}C_{pq}^{\left(1\right)}w_{q}^{*}+2{{\bar{w}}_{,\xi }^{*}|}_{\xi =\xi_{p}}{{\bar{w}}_{,\xi \xi }^{*}|}_{\xi =\xi_{p}}\right)]\}\\ \Lambda_{2}^{*}=D_{0}^{*}\alpha \;\{{{\bar{w}}_{,\xi }^{*}|}_{\xi =\xi_{p}} & \overset{R}{\underset{q=1}{\sum }}C_{pq}^{\left(4\right)}u_{q}^{*}+3{{\bar{w}}_{,\xi \xi }^{*}|}_{\xi =\xi_{p}}\overset{R}{\underset{q=1}{\sum }}C_{pq}^{\left(3\right)}u_{q}^{*}+3{{\bar{w}}_{,\xi \xi \xi }^{*}|}_{\xi =\xi_{p}}\overset{R}{\underset{q=1}{\sum }}C_{pq}^{\left(2\right)}u_{q}^{*}\\ & +{{\bar{w}}_{,\xi \xi \xi \xi }^{*}|}_{\xi =\xi_{p}}\overset{R}{\underset{q=1}{\sum }}C_{pq}^{\left(1\right)}u_{q}^{*}\\ & +\overset{R}{\underset{q=1}{\sum }}C_{pq}^{\left(4\right)}w_{q}^{*}[\overset{R}{\underset{q=1}{\sum }}C_{pq}^{\left(1\right)}u_{q}^{*}+\frac{3}{2}\alpha {\left(\overset{R}{\underset{q=1}{\sum }}C_{pq}^{\left(1\right)}w_{q}^{*}\right)}^{2}\\ & +3\alpha {{\bar{w}}_{,\xi }^{*}|}_{\xi =\xi_{p}}\overset{R}{\underset{q=1}{\sum }}C_{pq}^{\left(1\right)}w_{q}^{*}+{\left({{\bar{w}}_{,\xi }^{*}|}_{\xi =\xi_{p}}\right)}^{2}]\\ & +\overset{R}{\underset{q=1}{\sum }}C_{pq}^{\left(3\right)}w_{q}^{*}[3\overset{R}{\underset{q=1}{\sum }}C_{pq}^{\left(2\right)}u_{q}^{*}+9\alpha \overset{R}{\underset{q=1}{\sum }}C_{pq}^{\left(1\right)}w_{q}^{*}\overset{R}{\underset{q=1}{\sum }}C_{pq}^{\left(2\right)}w_{q}^{*}\\ & +9\alpha {{\bar{w}}_{,\xi }^{*}|}_{\xi =\xi_{p}}\overset{R}{\underset{q=1}{\sum }}C_{pq}^{\left(2\right)}w_{q}^{*}+9\alpha {{\bar{w}}_{,\xi \xi }^{*}|}_{\xi =\xi_{p}}\overset{R}{\underset{q=1}{\sum }}C_{pq}^{\left(1\right)}w_{q}^{*}\\ & +6\alpha {{\bar{w}}_{,\xi }^{*}|}_{\xi =\xi_{p}}{{\bar{w}}_{,\xi \xi }^{*}|}_{\xi =\xi_{p}}]\\ & +\overset{R}{\underset{q=1}{\sum }}C_{pq}^{\left(2\right)}w_{q}^{*}[3\overset{R}{\underset{q=1}{\sum }}C_{pq}^{\left(3\right)}u_{q}^{*}+3\alpha {\left(\overset{R}{\underset{q=1}{\sum }}C_{pq}^{\left(2\right)}w_{q}^{*}\right)}^{2}\\ & +9\alpha {{\bar{w}}_{,\xi \xi }^{*}|}_{\xi =\xi_{p}}\overset{R}{\underset{q=1}{\sum }}C_{pq}^{\left(2\right)}w_{q}^{*}+9\alpha {{\bar{w}}_{,\xi \xi \xi }^{*}|}_{\xi =\xi_{p}}\overset{R}{\underset{q=1}{\sum }}C_{pq}^{\left(1\right)}w_{q}^{*}+6\alpha {\left({{\bar{w}}_{,\xi \xi }^{*}|}_{\xi =\xi_{p}}\right)}^{2}\\ & +6\alpha {{\bar{w}}_{,\xi }^{*}|}_{\xi =\xi_{p}}{{\bar{w}}_{,\xi \xi \xi }^{*}|}_{\xi =\xi_{p}}]\\ & +\overset{R}{\underset{q=1}{\sum }}C_{pq}^{\left(1\right)}w_{q}^{*}[\overset{R}{\underset{q=1}{\sum }}C_{pq}^{\left(4\right)}u_{q}^{*}+\frac{3}{2}\alpha {{\bar{w}}_{,\xi \xi \xi \xi }^{*}|}_{\xi =\xi_{p}}\overset{R}{\underset{q=1}{\sum }}C_{pq}^{\left(1\right)}w_{q}^{*}\\ & +2\alpha {{\bar{w}}_{,\xi }^{*}|}_{\xi =\xi_{p}}{{\bar{w}}_{,\xi \xi \xi \xi }^{*}|}_{\xi =\xi_{p}}+6\alpha {{\bar{w}}_{,\xi \xi }^{*}|}_{\xi =\xi_{p}}{{\bar{w}}_{,\xi \xi \xi }^{*}|}_{\xi =\xi_{p}}]\} \end{array}$$

Boundary conditions at the end points of a hinged-hinged beam can be implemented using DQ discretization as

(25) $$\begin{array}{c} u_{𝓀}^{*}=w_{𝓀}^{*}=0\\ \begin{array}{cc} D_{2}^{*}\overset{R}{\underset{q=1}{\sum }}C_{𝓀q}^{\left(1\right)}\Phi_{q}^{*}- & D_{0}^{*}\mu^{2}[{\bar{w}}_{,\xi }^{*}\overset{R}{\underset{q=1}{\sum }}C_{𝓀q}^{\left(2\right)}u_{q}^{*}+{\bar{w}}_{,\xi \xi }^{*}\overset{R}{\underset{q=1}{\sum }}C_{𝓀q}^{\left(1\right)}u_{q}^{*}\\ & +\overset{R}{\underset{q=1}{\sum }}C_{𝓀q}^{\left(1\right)}w_{q}^{*}\left(\overset{R}{\underset{q=1}{\sum }}C_{𝓀q}^{\left(2\right)}u_{q}^{*}+\frac{3}{2}\alpha {\bar{w}}_{,\xi \xi }^{*}\overset{R}{\underset{q=1}{\sum }}C_{𝓀q}^{\left(1\right)}w_{q}^{*}+2\alpha {\bar{w}}_{,\xi \xi }^{*}{\bar{w}}_{,\xi }^{*}\right)\\ & +\overset{R}{\underset{q=1}{\sum }}C_{𝓀q}^{\left(2\right)}w_{q}^{*}(\overset{R}{\underset{q=1}{\sum }}C_{𝓀q}^{\left(1\right)}u_{q}^{*}+\frac{3}{2}\alpha \;{\left(\overset{R}{\underset{q=1}{\sum }}C_{𝓀q}^{\left(1\right)}w_{q}^{*}\right)}^{2}+3\alpha {\bar{w}}_{,\xi }^{*}\overset{R}{\underset{q=1}{\sum }}C_{𝓀q}^{\left(1\right)}w_{q}^{*}\\ & +\alpha {\bar{w}}_{,\xi }^{*}{\bar{w}}_{,\xi }^{*})]+I_{2}^{*}\mu^{2}\overset{R}{\underset{q=1}{\sum }}C_{𝓀q}^{\left(1\right)}{\ddot{\Phi }}_{q}^{*}-I_{0}^{*}\mu^{4}\overset{R}{\underset{q=1}{\sum }}C_{𝓀q}^{\left(2\right)}{\ddot{u}}_{q}^{*}\left(\overset{R}{\underset{q=1}{\sum }}C_{𝓀q}^{\left(1\right)}w_{q}^{*}+{\bar{w}}_{,\xi }^{*}\right)\\ & -I_{0}^{*}\mu^{4}\overset{R}{\underset{q=1}{\sum }}C_{𝓀q}^{\left(1\right)}{\ddot{u}}_{q}^{*}\left(\overset{R}{\underset{q=1}{\sum }}C_{𝓀q}^{\left(2\right)}w_{q}^{*}+{\bar{w}}_{,\xi \xi }^{*}\right)+\frac{1}{\alpha }\mu^{2}I_{0}^{*}{\ddot{w}}^{*}=0 \end{array} \end{array}$$

where

𝓀=1 at ξ=0 and 𝓀=R at ξ=1

Considering the displacement vector of each grid point as $d_{i}={\left\{u_{i}^{*},\;w_{i}^{*},\Phi_{i}^{*}\right\}}^{T}$ for i=1, ⋯,R, it is possible to write Equation (23) in the following form

(26) $${\left[K\right]}_{3R\times 3R}{\left\{X\right\}}_{3R\times 1}+{\left[M\right]}_{3R\times 3R}{\left\{\ddot{X}\right\}}_{3R\times 1}={\left\{0\right\}}_{3R\times 1}$$

where [K] is the stiffness matrix that can be decomposed into linear and nonlinear parts $\left(\left[K\right]={\left[K\right]}_{L}+{\left[K\right]}_{NL}\right)$. [M] is the mass matrix and $\left\{X\right\}\left(={\left\{d_{i}\right\}}^{T}\;,\;i=1,\cdots ,R\right)$ is the generalized vector of displacement components. A harmonic motion $\left\{X\right\}=\left\{\tilde{X}\right\}e^{i\omega^{*}t^{*}}$(where $\omega^{*}=\omega L\sqrt{I_{0}/EA}$ is the dimensionless frequency and ω is the vibration frequency of the nano-beam) is considered. By substitution of this expression for {X} in Equation (26) and using the decomposition of stiffness matrix, an eigenvalue equation will be obtained as

(27) $$\left({\left[K\right]}_{L}+{\left[K\right]}_{NL}\right)\left\{\tilde{X}\right\}-{\omega^{*}}^{2}\left[M\right]\left\{\tilde{X}\right\}=\left\{0\right\}$$

The iterative solution procedure to Equation (27) starts with ignoring the nonlinear part of the stiffness matrix and solving the corresponding linear eigenvalue problem. Thus it is possible to find eigenvalues and eigenvectors of each linear vibration mode. The vector of considered mode shape is appropriately scaled up in a way that the maximum component of lateral displacement is equal to a given dimensionless nonlinear vibration amplitude $w_{max}^{*}$. After that, using the modified eigenvector, the nonlinear part of stiffness matrix is calculated and a new eigenvalue problem is formed to solve for new eigenvalues and eigenvectors. The corresponding eigenvector of considered mode shape is again scaled up and the procedure continues until the relative difference between the eigenvalue of the current iteration and previous one is lower than a predefined value [34].

## 3. Numerical Results and Discussions

### 3.1. Results Verification

In this section, the values of fundamental frequency obtained by the present method are compared to those available for various cases in the literature. The number of sampling grid points for all the numerical results presented herein and in the next section is set to *R* = 11, which is the minimum value obtained by performing convergence studies for nonlinear vibration of a nonlocal Timoshenko nano-beam.

Table 1 presents the comparison between the results of nonlinear to linear fundamental frequency ratio $\left(\omega_{nl}/\omega_{l}\right)$ of a Timoshenko beam obtained by the present solution approach and those obtained by the finite element method [35,36]. Various values of dimensionless nonlinear vibration amplitude, $w_{\text{max}}/\sqrt{I_{2}/A}$, and different boundary conditions are considered. Beam geometrical and mechanical properties are given in Marur [36] and imperfection amplitude *η*, and nonlocal parameter *μ*, are set to zero. Close correlation between present iterative DQ approach and finite element method is achieved.

To maintain the confidence in results obtained based on nonlocal constitutive equations, the dimensionless linear frequencies ($\omega l^{2}\sqrt{\rho A/EI_{2}}$) of a straight (*η = 0*) SWCNT are listed in Table 2 for different values of the nonlocal parameter, and various boundary conditions. For possibility of comparison, the SWCNT properties are chosen as follows: radius *r* = 0.339 nm, tube thickness *h* = 0.066 nm, Young’s modulus *E* = 5.5 TPa, Poisson’s ratio *υ* = 0.19, and shear correction factor *K_s_* = 0.563 [37]. As expected, increasing the nonlocal effect causes a decrease in dimensionless frequency. A noteworthy agreement is observed between the present results and those reported by Yang et al. [22]. Good agreement is observed between the results of current study and those by Wang et al. [37]. However, because they neglected the nonlocal terms in shearing force relation, the discrepancy between the results of two methods increases for higher values of nonlocal parameter.

For the last verification case of the proposed method, Table 3 shows the linear and nonlinear dimensionless frequencies for a straight (*η* = 0) single-walled nanotube with *r =* 0.313 nm, *L* = 5 nm, *E* = 1.1556 TPa, *υ* = 0.19, *h* = 0.34 nm and *K_s_* = 0.563. Nonlocal parameter, μ, and nonlinear vibration amplitude, $w_{max}^{*}$, are set to 0.15 and 0.4, respectively. For both linear and nonlinear frequencies, good agreement exists between the results of current study and those reported in [22].

### 3.2. Geometric Imperfection Function

**Figure 2:**
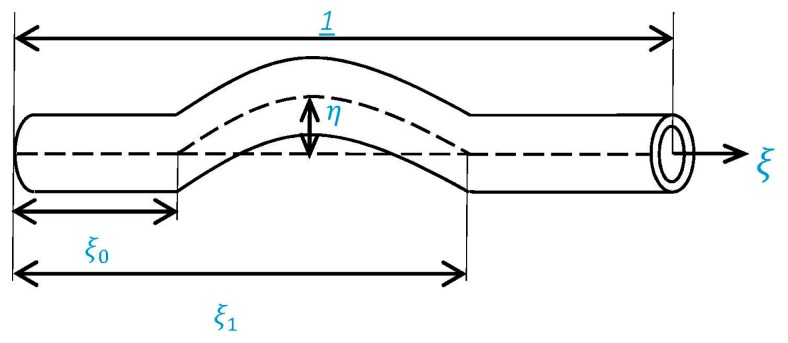
Schematic of initial imperfection of the nano-beam.

The following functional form of initial geometric imperfection is considered for the purpose of numerical computations:

(28) $$\begin{array}{ccc} {\bar{w}}^{*}=\eta \;\cos\frac{\pi \left(2\xi -\gamma -\beta \right)}{2\left(\gamma -\beta \right)} & & \xi_{0}\leq \xi \leq \xi_{1}\\ {\bar{w}}^{*}=0 & & \text{elsewhere} \end{array}$$

where η is the dimensionless amplitude of geometrical imperfection. Figure 2 shows the schematic of the imperfection function considered in this study.

**Figure 3:**
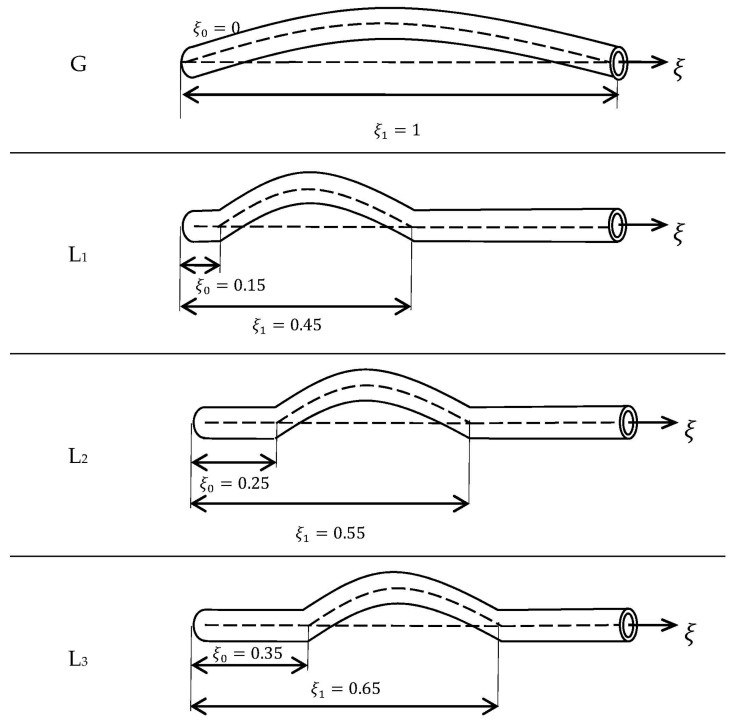
Various imperfection types considered in numerical results.

To be able to study the effects of location and extension of the geometric imperfection on the vibration characteristics of SWCNT, four cases as described in Figure 3 are considered. The “G” type represents a symmetrical full-length imperfection while the local imperfections with variable endpoint locations are denoted by “L_1_” to “L_3_” types. It is seen that the imperfection is exactly located at the center of SWCNT for L_3_ type while for the L_1_ type the imperfection is the closest case to the end.

### 3.3. Nonlinear Vibration of Curved SWCNTs

After verification of the formulation and solution procedure, in this section, the results for a SWCNT with Young’s modulus of E=1.1556 TPa and Poisson’s ratio ν=0.19 are presented. The geometrical properties are taken as: mean radius, r=0.5 nm, thickness of the tube h=0.3 nm and the total length, L=5 nm unless otherwise specified. The magnitude of shear correction factor for a tubular cross-section is given by $K_{s}=0.563$ [22]. Global and local initial geometric imperfections are considered as discussed in Section 3.2 for clamp-clamp (C–C), clamp-hinged (C–H) and hinged-hinged (H–H) boundary conditions. 

**Figure 4:**
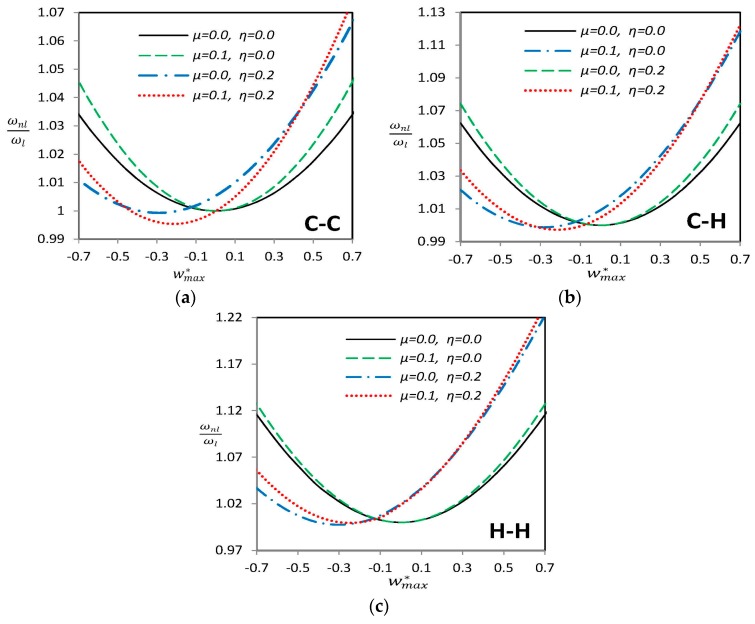
Variation of nonlinear vibration frequency ratio for different combinations of imperfection amplitude *η* and nonlocal parameter *μ* for a “G” type imperfection for (**a**) clamped-clamped (**b**) clamped-hinged and (**c**) hinged-hinged endpoint conditions.

Figure 4 demonstrates the nonlinear to linear frequency ratio ($\omega_{nl}/\omega_{l}$) versus maximum nonlinear vibration amplitude $w_{max}^{*}$ of a curved SWCNT with “G” type geometric imperfection with and without considering nonlocal parameter *μ*. Note that for all graphs, although $\omega_{nl}$ is the nonlinear frequency of the perfect or imperfect SWCNT, $\omega_{l}$ is the linear frequency of the perfect one. It can be observed that for perfect cases (*η* = 0), symmetry with respect to $w_{max}^{*}=0$ exists. Thus the beam shows the same vibrational characteristics for positive and negative values of vibration amplitude. This is not the case for an imperfect beam as Figure 4 illustrates. It is mainly because the initial geometric imperfection is not generally symmetric with respect to *x*-axis which annihilates the symmetry of the problem. The nonlinear to linear frequency ratio increases while the vibration amplitude $w_{max}^{*}$ is increased which is expected in large amplitude vibration of structures as a consequence of hard-spring behavior. Increasing nonlocal parameter *μ* causes a decrease in value of both linear and nonlinear frequencies. As Figure 4 indicates, the nonlinear to linear frequency ratio increases when the nonlocal parameter is increased, which means that this decrease in frequency value is more drastic for the linear frequency. It can also be observed that the rate of increase of frequency ratio is greater when a nonlocal parameter is present for both straight and curved SWCNTs.

**Figure 5:**
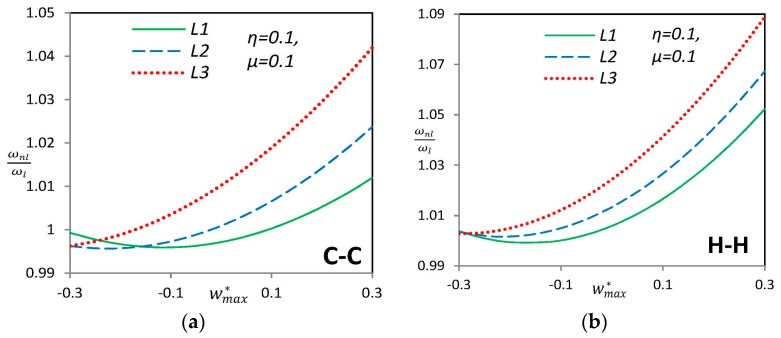
Variation of nonlinear vibration frequency ratio for (**a**) clamped-clamped and (**b**) hinged-hinged endpoint conditions of a nonlocal nano-beam for various “L” types imperfection.

The influence of the location of geometric imperfection on the nonlinear frequency ratio of curved SWCNTs with *μ* = 0.1 is presented in Figure 5 for H–H and C–C boundary conditions. “L_1_,” “L_2_” and “L_3_” are local imperfections with maximum amplitude of *η* = 0.1. For all local imperfect cases, the hard-spring behavior is observed. It is seen that the local imperfection has the largest effect on the nonlinear frequency ratio when it is located in the center of the single-walled carbon nanotube (L_3_) and its effect is decreased when the imperfection recedes from the center to the boundary (L_1_). 

**Figure 6:**
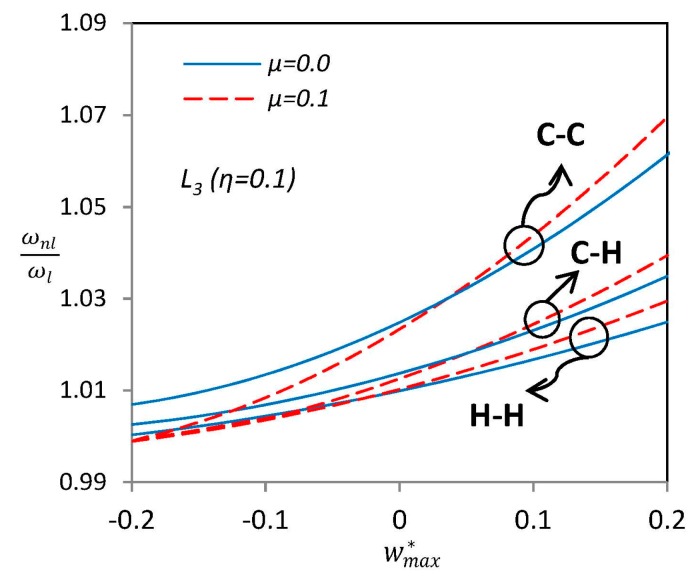
Effects of nonlocal parameter *μ* on the nonlinear vibration frequency ratio of nano-beam with “L_3_” type of initial imperfection.

Figure 6 focuses on the effect of nonlocal parameter on the nonlinear frequency ratio of SWCNTs. The local case “L_3_” is selected as the geometric imperfection with maximum amplitude of *η* = 0.1. Unlike straight SWCNTs (perfect case), curved nanotubes (imperfect cases) do not have an overall increasing trend for nonlinear frequency ratio while the nonlocal parameter *μ* raises, and there is a certain value of amplitude vibration $w_{max}^{*}$ in which the effect of nonlocal parameter gets reversed. Furthermore, it is observed that SWCNTs with C–C boundary condition have the highest nonlinear frequency and the H–H SWCNTs have the lowest, as expected.

**Figure 7:**
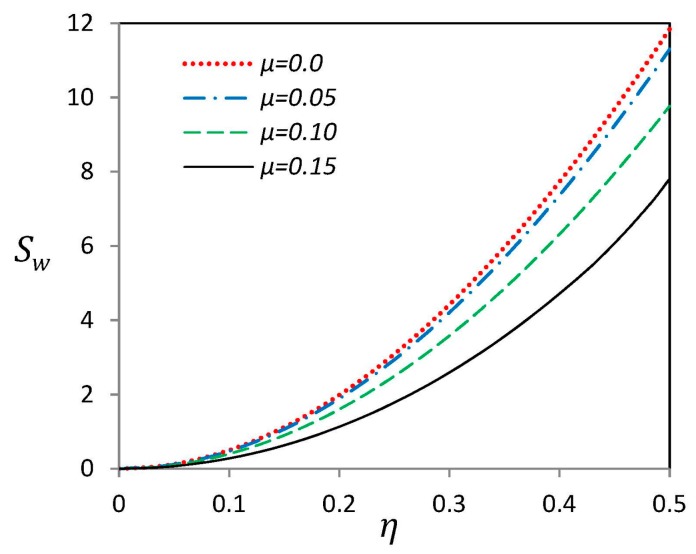
Effect of nonlocal parameter *μ* on sensitivity indicator of a nano-beam with “G” type initial imperfection with hinged-hinged endpoint conditions.

Figure 7 depicts imperfection sensitivity of the linear fundamental frequency of a H–H curved SWCNT with “G” type geometric imperfection for different values of nonlocal parameter *μ*. The imperfection sensitivity indicator is defined as [24]:

(29) $$S_{w}=\frac{\omega_{imperfect}-\omega_{perfect}}{\omega_{perfect}}\times 100\%$$

where $\omega_{perfect}$ and $\omega_{imperfect}$ are dimensionless fundamental linear frequencies of straight and curved SWCNTs, respectively. Indeed, imperfection sensitivity indicator, $S_{w}$, presents the percent of changes in the linear frequency because of existence of imperfections. For a constant value of nonlocal parameter *μ*, it is obvious that the imperfection sensitivity, $S_{w}$, increases as the imperfection amplitude *η* is increased. On the other hand, for a constant value of *η*, increasing the nonlocal parameter causes a decrease in imperfection sensitivity. It means that curved SWCNTs with higher value of nonlocal parameter *μ* are less sensitive to “G” type geometric imperfections. Besides, imperfection sensitivity indicator can demonstrate the quantity of error due to neglecting imperfections in modeling. For instance, for all values of nonlocal parameter and *η* < 0.2, imperfection sensitivity and therefore the error of neglecting imperfection of curved SWCNTs is less than 2%.

**Figure 8:**
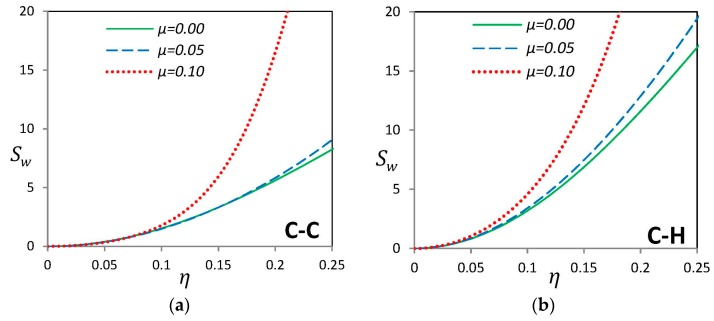

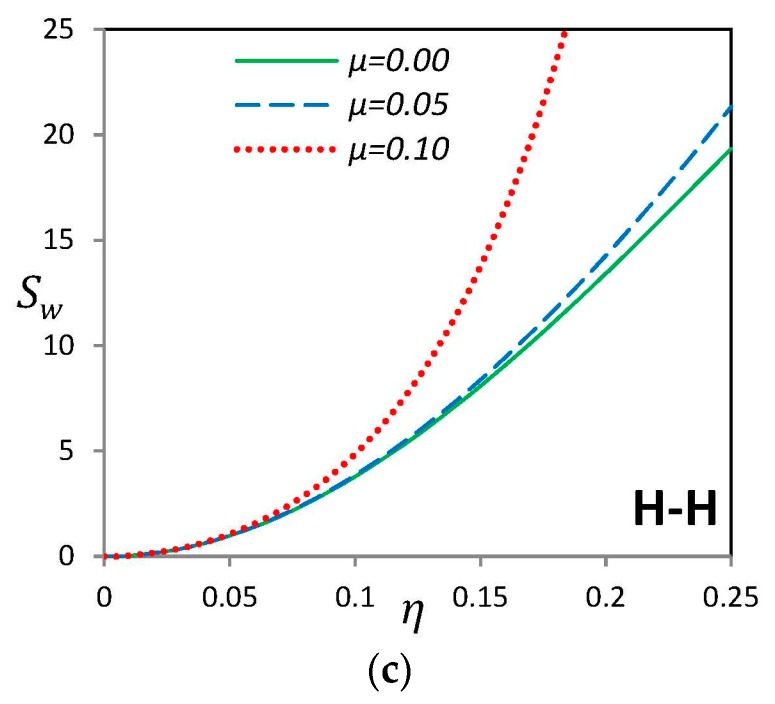
Effect of nonlocal parameter *μ* on sensitivity indicator of a nano-beam with “L_2_” type initial imperfection for (**a**) clamped-clamped (**b**) clamped-hinged and (**c**) hinged-hinged endpoint conditions.

Investigation on the imperfection sensitivity of the linear fundamental frequency of curved SWCNTs with local (L_2_) geometry is illustrated in Figure 8. Like the “G” type imperfection, the imperfection sensitivity $S_{w}$ increases when the maximum imperfection amplitude *η* is raised. For small values of imperfection amplitude *η*, all values of the nonlocal parameter almost have the same behavior, but after a certain value of *η*, a sudden large increase in imperfection sensitivity value occurs for higher values of nonlocal parameter *μ*. It depicts that for the case of local imperfection with large values of nonlocal parameter, initial geometric imperfections may cause considerable change in the fundamental frequency which cannot be neglected.

## 4. Conclusions

In the present study, based on the nonlocal theory of Eringen, nonlinear vibration of curved SWCNTs with various boundary conditions has been investigated through the DQ solution approach. Curved SWCNTs are considered as a Timoshenko beam model with global and local initial geometric imperfections. Numerical results for a curved SWCNT indicate that the value and the location of geometric imperfection have considerable effect on the linear and nonlinear vibration characteristics of curved nano-beams. Higher imperfection amplitudes clearly increase the vibration frequency of the nano-beam. Furthermore, centrally located geometric imperfections have the largest influence on increasing the frequency ratios of curved SWCNTs. On the other hand, a small-scale parameter may also increase or decrease the nonlinear frequency ratio and sensitivity indicator depending on the amplitude, type and location of the imperfection of the nano-beam.

## Figures and Tables

**Table 1:** Comparison of nonlinear frequency ratio of a Timoshenko straight beam for different vibration amplitudes.

$\frac{w_{\text{max}}}{\sqrt{I_{2}/A}}$	H–H			C–C			C–H		
	Ref. [35]	Ref. [36]	Present	Ref. [35]	Ref. [36]	Present	Ref. [35]	Ref. [36]	Present
1.0	1.118	1.118	1.1181	1.0295	1.0283	1.0295	1.0641	1.0582	1.0593
2.0	1.4141	1.4135	1.4143	1.1127	1.1105	1.1128	1.2318	1.215	1.2182
3.0	1.8026	1.8027	1.8029	1.2377	1.2336	1.2378	1.4603	1.4368	1.4416
4.0	2.2359	2.2361	2.2363	1.3920	1.3856	1.3921	1.7210	1.6822	1.7026
5.0	2.6923	2.6925	2.6928	1.5659	1.5574	1.5660	1.9995	1.9180	1.9862

**Table 2:** Comparison of dimensionless linear frequency ($\omega l^{2}\sqrt{\rho A/EI_{2}}$) of a straight nano-beam with different nonlocal parameter *µ* and various endpoint conditions.

*μ*	H–H			C–C			C–H		
	Ref. [37]	Ref. [22]	Present	Ref. [37]	Ref. [22]	Present	Ref. [37]	Ref. [22]	Present
0.0	3.0929	–	3.0929	4.4491	–	4.4491	3.7845	–	3.7844
0.1	3.0243	3.0210	3.0210	4.3471	4.3269	4.3269	3.6939	3.6849	3.6849
0.3	2.6538	2.6385	2.6385	3.7895	3.7032	3.7032	3.2115	3.1724	3.1724
0.5	2.2867	2.2665	2.2665	3.2420	3.1372	3.1371	2.7471	2.6982	2.6980
0.7	2.0106	–	1.9899	2.8383	–	2.7327	2.4059	–	2.3569

**Table 3:** Comparison of linear and nonlinear dimensionless frequency of a straight nonlocal nano-beam with *μ* = 0.15 and $w_{max}^{*}=0.4$.

Frequency	H–H		C–C		C–H	
	Ref. [22]	Present	Ref. [22]	Present	Ref. [22]	Present
$\omega_{l}^{*}$	0.4233	0.4233	0.8055	0.8055	0.6052	0.6052
$\omega_{nl}^{*}$	0.4405	0.4435	0.8188	0.8219	0.6197	0.6236

## Abbreviations

The following abbreviations are used in this manuscript:

CNTs	Carbon Nano Tubes
DQ	Differential Quadrature
MD	Molecular Dynamics
NEMS	Nano Electro Mechanical System
SWCNTs	Single-Walled Carbon Nano Tubes
